# Cadmium Alters the Metabolism and Perception of Abscisic Acid in *Pisum sativum* Leaves in a Developmentally Specific Manner

**DOI:** 10.3390/ijms25126582

**Published:** 2024-06-14

**Authors:** Edyta Zdunek-Zastocka, Beata Michniewska, Angelika Pawlicka, Agnieszka Grabowska

**Affiliations:** Department of Biochemistry and Microbiology, Institute of Biology, Warsaw University of Life Sciences—SGGW, Nowoursynowska 159, 02-776 Warsaw, Polands200057@sggw.edu.pl (A.P.);

**Keywords:** abscisic acid, ABA receptors, abscisic acid-glucosyl esters, aldehyde oxidase, cadmium stress, 9-cis-epoxycarotenoid dioxygenase, β-glucosidase, pyrabactin resistance-like (PYR/PYL) proteins

## Abstract

Abscisic acid (ABA) plays a crucial role in plant defense mechanisms under adverse environmental conditions, but its metabolism and perception in response to heavy metals are largely unknown. In *Pisum sativum* exposed to CdCl_2_, an accumulation of free ABA was detected in leaves at different developmental stages (A, youngest, unexpanded; B1, youngest, fully expanded; B2, mature; C, old), with the highest content found in A and B1 leaves. In turn, the content of ABA conjugates, which was highest in B2 and C leaves under control conditions, increased only in A leaves and decreased in leaves of later developmental stages after Cd treatment. Based on the expression of *PsNCED2*, *PsNCED3* (9-cis-epoxycarotenoid dioxygenase), *PsAO3* (aldehyde oxidase) and *PsABAUGT1* (ABA-UDP-glucosyltransferase), and the activity of PsAOγ, B2 and C leaves were found to be the main sites of Cd-induced de novo synthesis of ABA from carotenoids and ABA conjugation with glucose. In turn, β-glucosidase activity and the expression of genes encoding ABA receptors (*PsPYL2*, *PsPYL4*, *PsPYL8*, *PsPYL9*) suggest that in A and B1 leaves, Cd-induced release of ABA from inactive ABA-glucosyl esters and enhanced ABA perception comes to the forefront when dealing with Cd toxicity. The distinct role of leaves at different developmental stages in defense against the harmful effects of Cd is discussed.

## 1. Introduction

Environmental pollution, driven mainly by rapid industrialization and extensive agriculture, is a major challenge for the world today. Heavy metals are among the most hazardous pollutants as they are toxic, non-biodegradable and tend to accumulate in living organisms [1]. Cadmium (Cd), a non-essential element with no known biological function in living organisms, is particularly toxic [2]. It is easily absorbed by plants through the roots and transported to the leaves via the xylem [3]. The high affinity of Cd for sulfhydryl groups in proteins and its ability to replace essential metals in the active sites of enzymes make it extremely toxic even at low concentrations. In addition, Cd causes a decrease in chlorophyll content, inhibits the process of photosynthesis and transpiration [4], reduces the uptake of the mineral nutrients and impairs their transport [5]. Although Cd is not a redox-active metal, it can trigger oxidative stress by causing the overproduction and accumulation of reactive oxygen species (ROS), which in turn lead to lipid peroxidation and damage to cell membranes and organelles [6]. Visible symptoms of Cd toxicity include chlorosis, necrosis, growth inhibition and even plant death, at high concentrations [7].

To counteract the harmful effects of stress factors such as cadmium, plants have developed several defense mechanisms, of which abscisic acid (ABA) plays a central role. ABA is a phytohormone involved in plant growth and development, including the promotion of senescence, induction of seed dormancy and closure of stomata [8]. Under appropriate environmental conditions, adequate ABA concentration enables optimal plant growth, while ABA, which rapidly accumulates in plant tissues under various stress factors, plays a protective role [9,10,11]. ABA has been found to increase plant tolerance to cadmium by reducing transpiration and thus reducing Cd uptake from the soil and transport from roots to shoots [3,12,13]. Moreover, ABA promotes the activity of antioxidant enzymes and increases the content of non-enzymatic antioxidants [14]. Furthermore, ABA induces phytochelatin-conjugated vacuolar sequestration of Cd and inhibits the expression of genes encoding metal transporters [15].

Strict control of ABA levels is crucial for proper plant growth and development, and for adaptation to unfavorable environmental conditions. The concentration of ABA is regulated in tissues by its synthesis, degradation, (de)conjugation and transport [9]. In plants, abscisic acid is synthesized de novo via the carotenoid pathway, which starts with the cleavage of β-carotene [16]. A key enzyme of the ABA biosynthetic pathway that determines its rate in response to stress factors is 9-*cis*-epoxycarotenoid dioxygenase (NCED, EC 1.13.11.51) [16]. Overexpression of the *Vitis amurensis* gene encoding NCED resulted in increased tolerance to drought and flooding stress [17]. In addition, plants overexpressing *VaNCED1* genes also showed increased expression of other genes related to drought stress response, such as *ABA-responsive element 1* (*ABRE1*), *ABRE binding factors 2* and *ABA-insensitive 5*. In peas, increased expression of *PsNCED2* and *PsNCED3* was observed in both leaves and roots under progressive and rapid-onset drought stress [18]. The final step of the pathway, the oxidation of abscisic aldehyde (ABAld) to abscisic acid, is catalyzed by the molybdenum enzyme aldehyde oxidase (AO, EC 1.2.3.1). AO exhibits a wide range of substrate specificity [19]; however, AO isoforms that oxidize ABAld to ABA are only reported for Arabidopsis (AAOδ) [20], barley (AO3) [21] and pea (PsAOγ) [22]. PsAOγ is a homodimer consisting of subunits encoded by the *PsAO3* gene [18]. Importantly, PsAOγ exhibits enhanced activity towards ABAld in both leaves and roots under drought [18], salt and ammonium treatments [22]. The degradation of abscisic acid is based on its conversion to phaseic acid (PA), which is catalyzed by a cytochrome P450 monooxygenase (P450, EC 1.14.14.1) [23]. However, the level of free active ABA is also controlled by its conjugation. ABA-UDP-glucosyltransferase (ABAUGT, EC 2.4.1.163) catalyzes the glucosylation that leads to the formation of the inactive storage form of ABA, ABA-glucose ester (ABA-GE) [24]. In peas, ABAUGT is encoded by *PsABAUGT1* and *PsABAUGT2*, and overexpression of *PsABAUGT1* in *Arabidopsis thaliana* decreases the concentration of free ABA and accelerates cotyledon emergence [25]. Among the conjugates, ABA-GE stands out as the predominant form. When environmental conditions become unfavorable, β-glucosidase (BGLU, ABA-GE hydrolase, EC 3.2.1.175) rapidly converts ABA-GE to its active form [12,26,27]. ABA-GE is also considered as a transport form of abscisic acid for both local and long-distance transport through the xylem [9,28].

The response of plants to abscisic acid depends on the perception of signals mediated mainly through the pyrabactin resistance (PYR), pyrabactin resistance-like (PYL) and regulatory component of ABA receptors (RCAR) [29]. In the presence of ABA, the binding of PYR/PYL/RCAR to phosphatase 2C (PP2C) is enhanced, leading to inhibition of its activity [30,31]. This in turn abolishes PP2C-induced inhibition of sucrose non-fermenting 1-related protein kinase 2 (SnRK2), which subsequently targets specific membrane ion channels and transcription factors that are critical for adaptation to abiotic stress [32]. The Arabidopsis genome encodes 14 small RCAR/PYR1/PYL proteins (159–211 amino acid residues) that have different ABA-binding properties and selectively interact with PP2Cs to jointly regulate the ABA response [33,34]. Overexpression of *PYL5*, *PYL8*, *PYL9* and *PYL11-14* increases drought tolerance [35,36,37], while overexpression of *PYL12-13* improves tolerance to heat and cold stress [38]. In addition, the expression of individual *PYLs* depends on the tissue type, developmental phase and growth stage of the plant, and varies in response to environmental stimuli [39], contributing to the versatility of ABA signaling and functions.

There is still a lack of research focusing on the metabolism and perception of ABA in response to heavy metals, especially in leaves at different developmental stages. In this study, the changes in free ABA and ABA-GE content were investigated in younger and older pea leaves treated with cadmium. Furthermore, the expression of genes involved in the de novo synthesis of ABA and its conjugation with glucose was determined. This study also covered the activity of aldehyde oxidase and β-glucosidase as well as changes in the expression of *PYLs* in response to cadmium and exogenous ABA. The results obtained provide insight into how leaves prioritize and control their responses to cadmium stress at different developmental stages, which is crucial for the development of targeted strategies to increase plant resistance.

## 2. Results

### 2.1. Content of Free ABA and Its Conjugates as Affected by Cd Stress

The concentration of free ABA and its conjugates was analyzed in the leaves of pea plants after 48 h of growth on media containing 50 µM CdCl_2_. The analyses were performed on leaves at different stages of development.

**Figure 1:**
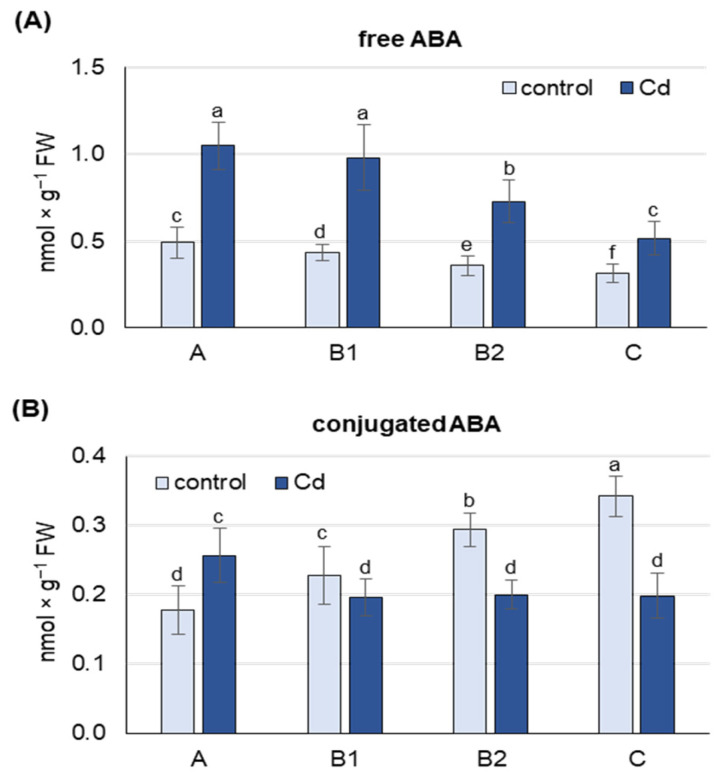
Changes in the concentration of free abscisic acid (**A**) and its conjugates (**B**) after 48 h of Cd treatment. Cadmium was applied as 50 µM CdCl_2_. A, the youngest unexpanded leaves; B1, the youngest fully expanded leaves; B2, fully expanded mature leaves; C, the oldest leaves. Results are the means (±SD) of three biological replicates. Different letters above the columns indicate significant differences between the means (*p* < 0.05).

Under both control and Cd stress conditions, the highest concentration of free ABA was found in the youngest, unexpanded A leaves, while B1, B2 and C leaves contained about 10, 25 and 40% less ABA, respectively (Figure 1A). After 48 h of Cd exposure, an increase in free ABA content was observed in leaves at each developmental stage, and this increase was about 2-fold in A, B1 and B2 leaves, and 1.5-fold in C leaves compared to control plants.

Under control conditions, the concentration of conjugated ABA forms was highest in the oldest C leaves, while B2, B1 and A leaves contained 15, 35 and 50% less ABA-GE, respectively (Figure 1B). This trend changed under Cd stress conditions, where the highest concentration of conjugated ABA forms was observed in A leaves and was 25 and 35% lower in B2, B1 and C leaves, respectively. Cd treatment increased the content of conjugated ABA forms, but only in the youngest A leaves (by 50%), while decreases of 15, 35 and 50% were observed in B1, B2 and C leaves, respectively.

### 2.2. Expression of Genes Involved in De Novo ABA Synthesis and ABA Conjugation with Glucose as Affected by Cd Stress

The transcript levels of genes encoding enzymes involved in de novo ABA synthesis (*PsNCED2*, *PsNCED3*, *PsAO3*) and ABA conjugation with glucose (*PsABAUGT1*) were analyzed in the leaves of pea plants after 48 h of growth on media containing 50 µM CdCl_2_. The analyses were performed on leaves at different stages of development.

**Figure 2:**
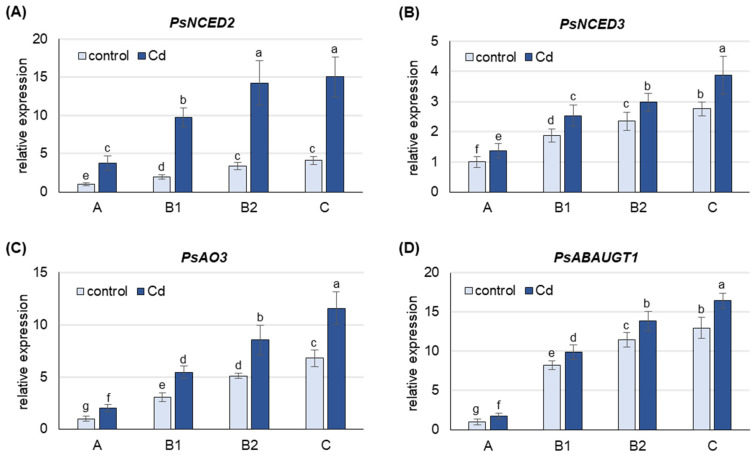
Changes in transcript levels of genes involved in ABA metabolism after 48 h of Cd treatment. (**A**) *PsNCED1*, (**B**) *PsNCED2*, (**C**) *PsAO3*, (**D**) *PsABAUGT1*. Cadmium was applied as 50 µM CdCl_2_. The relative mRNA level in each group of leaves was expressed relative to that in A leaves from control plants, which were set to 1 after normalizing to the reference gene. A, the youngest unexpanded leaves; B1, the youngest fully expanded leaves; B2, fully expanded mature leaves; C, the oldest leaves. Results are the means (±SD) of three biological replicates. Different letters above the columns indicate significant differences between the means (*p* < 0.05).

Under both control and Cd stress conditions, the mRNA level of all *Pisum sativum* genes analyzed was highest in the oldest C leaves, lower in B2 and B1 leaves and lowest in A leaves (Figure 2). Under control conditions, the expression of *PsNCED2*, *PsNCED3*, *PsAO3* and *PsABAUGT1* was approximately 4-, 3-, 6- and 13-fold higher in C leaves than in A leaves, respectively, and this trend was also observed in the Cd-treated plants. 

Treatment of the plants with CdCl_2_ resulted in an increase in the mRNA level of all genes analyzed, with the largest increase observed for *PsNCED2* (Figure 2). The expression of *PsNCED2*, *PsNCED3* and *PsAO3* was 4-, 1.5- and 2-fold higher, respectively, in Cd-treated plants than in control plants in leaves at each developmental stage. Cd also induced the expression of *PsABAUGT1*, which was increased by 75% in A leaves and 20–25% in the other leaves.

### 2.3. Activity of Aldehyde Oxidase and ABA-GE Hydrolase as Affected by Cd Stress

The activity of *Pisum sativum* aldehyde oxidase (PsAO), which catalyzes the final step of de novo ABA synthesis, and *Pisum sativum* β-glucosidase (PsBGLU, ABA-GE hydrolase), which releases ABA from its glucose conjugates, was investigated in leaves of pea plants after 48 h of growth on media containing 50 µM CdCl_2_. The analyses were carried out on leaves at different stages of development.

**Figure 3:**
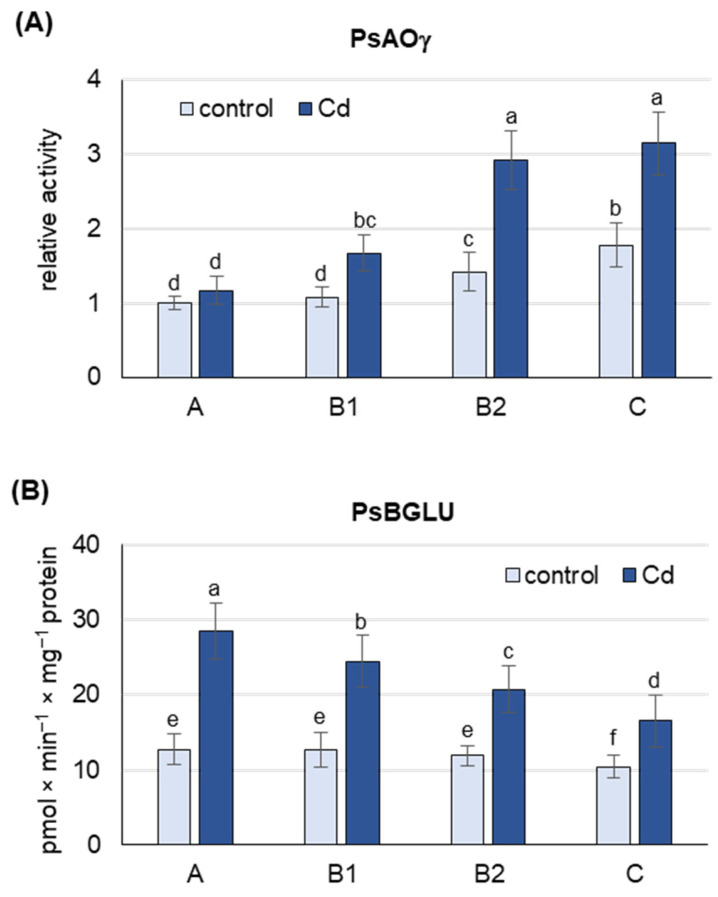
Activity of *Pisum sativim* aldehyde oxidase (PsAOγ) and β-glucosidase (PsBGLU, ABA-GE hydrolase) in leaves of the pea plants after 48 h of Cd treatment. Cadmium was applied as 50 µM CdCl_2_. (**A**) AO activity was assayed after native PAGE with indole-3-aldehyde as substrate. Each lane of the gel was loaded with 100 µg of proteins. The relative intensity of the PsAOγ isoform activity band in each group of leaves was quantified using ImageJ 1.53e software and expressed relative to that in A leaves from control plants set to 1. A representative zymogram is shown in Figure S2. (**B**) ABA-GE hydrolase activity was assayed using ABA-GE as substrate. A, the youngest unexpanded leaves; B1, the youngest fully expanded leaves; B2, fully expanded mature leaves; C, the oldest leaves. Results are the means (±SD) of three biological replicates. Different letters above the columns indicate significant differences between the means (*p* < 0.05).

Under both control and Cd stress conditions, the activity of PsAOγ, an isoform previously shown to oxidize abscisic aldehyde to ABA [22] and encoded by *PsAO3* [18], was highest in B2 and C leaves (Figure 3A and Figure S2). Under control conditions, the activity of PsAOγ was about 75% higher in B2 and C leaves than in A and B1 leaves. Cd treatment increased the activity of PsAOγ in B2 and C leaves by about 65%, but only by 15% in A leaves. Under Cd stress, the activity of PsAOγ in B2 and C leaves was already 2 and 2.5 times higher, respectively, than in A leaves, suggesting that B2 and C leaves are the major de novo ABA producers.

Under control conditions, the activity of PsBGLU was similar in A, B1 and B2 leaves and about 20% lower in C leaves (Figure 3B). Cd treatment increased PsBGLU activity in leaves of all developmental stages, with the most pronounced changes observed in A leaves and B1 (more than 2-fold increase). In C leaves of Cd-treated plants, PsBGLU activity was only 50% higher than in control plants. The results obtained suggest that Cd-induced ABA release from inactive ABA-glucosyl esters occurs mainly in A and B1 leaves.

### 2.4. Phylogenetic Analyses and Expression Levels of Genes Encoding ABA Receptors 

**Figure 4:**
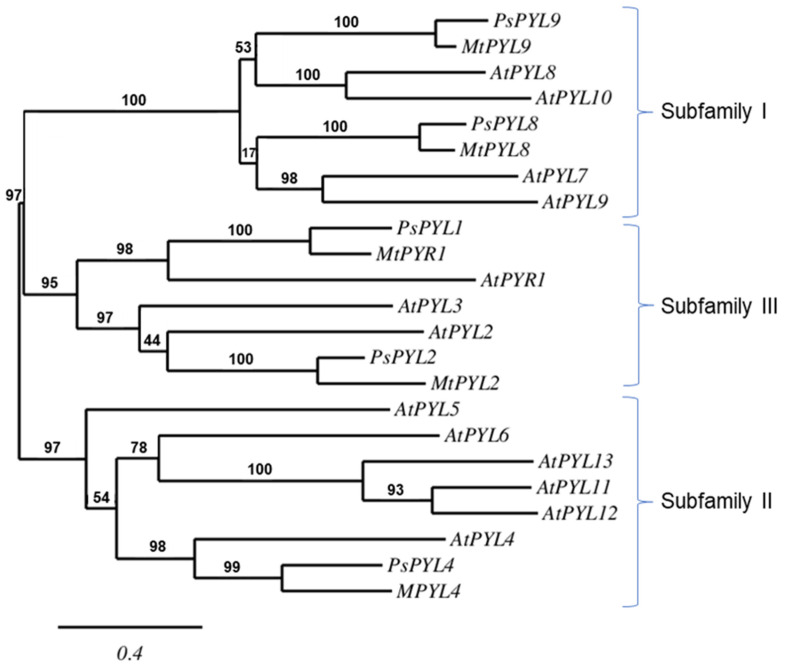
The phylogenetic relationship between the *PYLs* of *Arabidopsis thaliana* (At), *Medicago truncatula* (Mt) and *Pisum sativum* (Ps). The maximum likelihood tree was constructed using PhyML 3.0 software and the Shimodaira-Hasegawa-like (SH-like) test. The tree was displayed using TreeDyn. Numbers above branches indicate bootstrap support values after 100 replicates.

A database search on the NCBI server yielded five PYL sequences of *Pisum sativum*: *PsPYL1*, *PsPYL2*, *PsPYL4*, *PsPYL8*, *PsPYL9*. Based on the phylogenetic relationships, the *PsPYLs* were grouped into three *PYL* subfamilies together with the corresponding sequences of *Arabidospsis thaliana* and *Medicago truncatula*. *PsPYL1* and *PsPYL2* were assigned to subfamily III, *PsPYL4* to subfamily II and *PsPYL8* and *PsPYL9* to subfamily I (Figure 4).

The transcript levels of genes encoding ABA receptors were analyzed in leaves of pea plants after 48 h of growth on media containing 50 µM CdCl_2_. The analyses were performed on leaves at different stages of development. 

**Figure 5:**
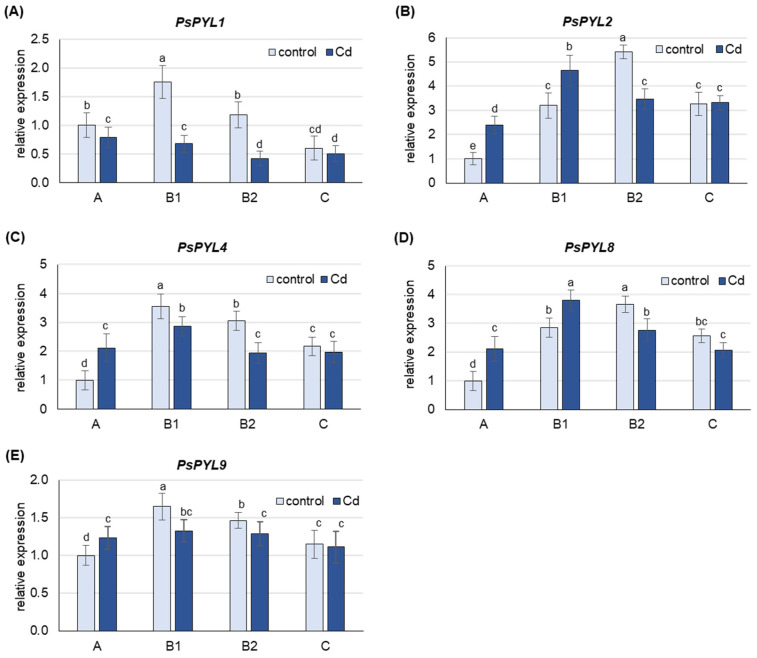
Changes in the transcript level of *PsPYLs* after 48 h of Cd treatment. Cadmium was applied as 50 µM CdCl_2_. (**A**) *PsPYL1*, (**B**) *PsPYL2*, (**C**) *PsPYL4*, (**D**) *PsPYL8*, (**E**) *PsPYL9*. The relative mRNA level in individual leaves was expressed relative to that in A leaves of control plants set to 1 after being normalized to the reference gene. A, the youngest unexpanded leaves; B1, the youngest fully expanded leaves; B2, fully expanded mature leaves; C, the oldest leaves. Results are the means (±SD) of three biological replicates. Different letters above the columns indicate significant differences between the means (*p* < 0.05).

Under control conditions, the highest level of *PsPYL* transcripts was observed in B1 (*PsPYL1*, *4*, *9*) and B2 (*PsPYL2*, *8*) leaves (Figure 5). The expression of *PsPYLs* in A leaves was lower than in B1 or B2 leaves, about 60% for *PsPYL1* and *PsPYL9*, more than 3-fold for *PsPYL4* and *PsPYL8* and 5-fold for *PsPYL2*. The expression of *PsPYLs* in C leaves was also lower than the corresponding highest level observed in B1 or B2 leaves, by 3-fold for *PsPYL1* and about 35% for the other *PsPYLs*.

Under Cd stress, an increase in the mRNA levels of *PsPYLs* was observed, but only in A leaves, where the expression of *PsPYL2*, *PsPYL4* and *PsPYL8* was more than 2-fold higher than in control plants, and in B1 leaves, where an increase of about 40% was observed for *PsPYL2* and *PsPYL8* (Figure 5). The expression of none of the *PsPYL* genes examined was increased by Cd in B2 and C leaves. On the contrary, Cd induced a significant decrease in the expression of all *PsPYL* genes in B2 leaves (by 30–200%), while only a slight decrease or no change in the transcript level of the analyzed genes was observed in C leaves.

### 2.5. Expression of Genes Encoding ABA Receptors as Affected by ABA Treatment

The transcript levels of genes encoding ABA receptors (*PsPYL1*, *PsPYL2*, *PsPYL4*, *PsPYL8*, *PsPYL9*) were analyzed in leaves of pea plants after 24 h of growth on media containing 50 µM ABA. The analyses were performed on leaves at different developmental stages. 

**Figure 6:**
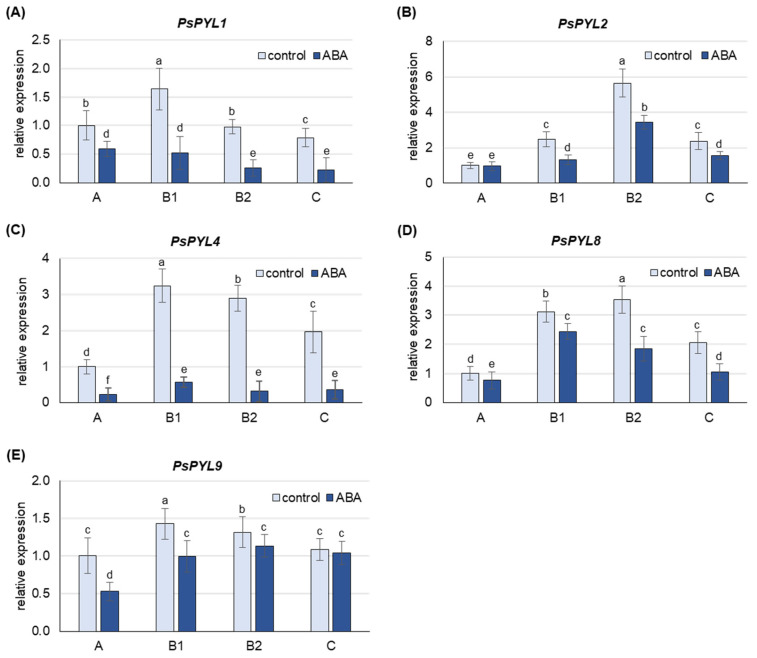
Changes in the transcript level of *PsPYLs* after 24 h of treatment with 50 µM ABA. (**A**) *PsPYL1*, (**B**) *PsPYL2*, (**C**) *PsPYL4*, (**D**) *PsPYL8*, (**E**) *PsPYL9*. The relative mRNA level in individual leaves was expressed in relation to that in A leaves of control plants, set to 1, after normalization to reference gene. A, the youngest unexpanded leaves; B1, the youngest fully expanded leaves; B2, fully expanded mature leaves; C, the oldest leaves. The results are the means (±SD) of three biological replicates. Different letters above the columns indicate significant differences between the means (*p* < 0.05).

ABA treatment increased the content of free ABA more than 3-fold in A leaves, 4- to 5-fold in B1 and B2 leaves and 8-fold in C leaves (Figure S3). At the same time, a down-regulation of the expression of all *PsPYLs* analyzed was also observed in the leaves of the individual developmental stages (Figure 6). The greatest decrease in mRNA levels was observed for *PsPYL4* and *PsPYL1*, where transcript levels were 5- to 10-fold and 2- to 4-fold lower, respectively, in ABA-treated plants compared to control plants. The least sensitive to ABA treatment was the expression of *PYL9*, which decreased approximately 2-fold in the youngest A leaves and 5–30% in leaves at other developmental stages.

## 3. Discussion

### 3.1. Cd Stress Increases the Concentration of Free ABA and Its Conjugates Mainly in the Youngest Leaves of Pea Plants

Under stress conditions, plants prioritize the protection of the youngest leaves, which are thought to make a greater contribution to plant fitness due to their relatively higher photosynthetic potential as a result of an unimpaired photosynthetic apparatus [40,41,42] and higher nitrogen content [43]. Younger leaves of *Barbarea* have been shown to contain higher concentrations of protective glucosinolates and saponins than older leaves when exposed to the herbivore *Plutella xylostella* [44]. It has also been reported that young leaves of Arabidopsis show a stronger antioxidant response than older leaves when exposed to drought [45] and herbicides [46]. Previous studies have also shown increased levels of other protective compounds, such as proline, especially in the youngest pea leaves when exposed to cadmium [47]. In monocotyledonous plants, the deleterious effects of Cd were observed to be less severe in the younger part of the leaves of rye [48] and maize [49].

Cadmium, an element known for its toxicity even at trace amounts, poses a significant threat to plants, forcing them to develop defense mechanisms to counter its negative effects. Abscisic acid is the main phytohormone involved in the plant’s response and tolerance to stress. Under Cd conditions, exogenous ABA significantly improves the growth characteristics of *Brassica napus* and *Lactuca sativa* plants, as measured by the accumulation of fresh biomass and the length of shoots and roots [50,51]. Exogenous ABA also mitigates the negative effects of Cd toxicity by increasing the content of chlorophyll a, chlorophyll b and carotenoids, improving gas exchange in the leaf and regulating the expression of genes encoding antioxidant enzymes and Cd stress-related genes [50,51]. In addition, ABA is known to reduce the transpiration rate by closing stomata and ultimately inhibiting Cd translocation into shoots [15].

An increase in the content of free endogenous ABA in leaves in response to cadmium exposure has been found in several plant species, including *Oryza sativa* [52], *Zea mays* [53], *Phaseolus vulgaris* [54] and *Mentha crispa* [55]. However, the changes in ABA content related to its metabolism in response to Cd in leaves at different developmental stages have not been investigated so far. In this study, it was observed that in the control plants, the highest free ABA content occurred in the youngest leaves, and gradually decreased with the stage of leaf development (Figure 1A). After a 48 h treatment with 50 µM cadmium, the ABA content doubled in the youngest A leaves and the fully developed B1 and B2 leaves, while a less pronounced increase was observed in the oldest C leaves. Similar results were obtained in previous studies on pea plants exposed to a 12 h treatment with Cd [47], where the highest free ABA content was also observed in young A and B1 leaves, both under control and stress conditions. The content of ABA conjugates showed an inverse trend, with the lowest levels found in the youngest A leaves and the highest levels in the oldest leaves of the control plants (Figure 1B). In turn, after Cd treatment, the content of ABA conjugates increased only in the youngest unexpanded A leaves and decreased in the leaves of later developmental stages, especially in the oldest C leaves. An increase in ABA-GE content in leaves of *Betula pendula* [56], *Cistus albidus* [57] and *Hordeum vulgare* [58] was found in response to drought; however, the response to stress in tissues at different developmental stages was not investigated. In this study, older leaves after Cd treatment showed a remarkable decrease in the content of ABA conjugates without a corresponding sharp increase in free ABA content, whereas younger leaves showed a marked increase in both ABA conjugate and free ABA content. As is commonly understood, the vast majority of ABA conjugates are ABA-GE, which serves as a storage and transport form of ABA, so that a transport of ABA-GE from the older to the younger leaves cannot be excluded.

### 3.2. Cd-Induced De Novo Synthesis of ABA and Its Conjugation with Glucose Occurs Mainly in Mature B2 and Old C Leaves, Whereas Its Release from Conjugates Occurs in Young A and B1 Leaves 

To gain a deeper insight into the rationale behind the increase in free ABA, the expression of genes specific for de novo ABA synthesis was examined (Figure 2). Interestingly, the increase in free ABA in response to Cd was highest in the youngest leaves, while the transcript levels of *PsNCED2*, *PsNCED3* and *PsAO3* genes, which encode key enzymes for de novo ABA synthesis, were lowest in these leaves, even in response to Cd. The expression of all genes tested showed a positive correlation with the stage of leaf development, with a further increase in transcript levels observed in response to Cd treatment. Furthermore, expression of *PsNCED2* increased more than 3-fold in mature B2 leaves and the oldest C leaves in response to cadmium. An increase in endogenous ABA levels associated with the up-regulation of *SaNCED* and *BnNCED3* was previously reported for roots of the non-hyperaccumulating ecotype *Sedum alfredii* [59] and leaves of *Boehmeria nivea* [60], respectively, after Cd exposure. In turn, an increase in *OsNCED4* expression was observed in *Oryza sativa* under Cd stress [61], while arsenic treatment strongly increased *OsNCED2* and *OsNCED3* expression [62]. In pea plants exposed to Cd, the highest expression of *PsNCEDs* and *PsAO3* observed in B2 and C leaves was accompanied by the highest activity of the PsAOγ isoform (Figure 3A). Indeed, the activity of the PsAOγ isoform, whose subunits are encoded by *PsAO3*, increased more in older leaves than in younger leaves in response to Cd exposure. It is noteworthy that, the observed increase in PsAOγ activity in the A leaves was comparatively modest. In previous studies, the most significant increase in PsAOγ activity was also observed in the oldest leaves of pea plants under gradually induced drought [18] and under prolonged treatments with salinity, low nitrogen and ammonium [22,63]. Since de novo ABA synthesis occurs predominantly in the B2 and C leaves of pea plants exposed to Cd, it is very likely that the remarkable increase in ABA concentration in the youngest A leaves is due to its transport from the older leaves. In addition, analysis of ABA-GE hydrolase activity (Figure 3B) revealed that under cadmium exposure, the release of ABA from ABA-GE occurs predominantly in the youngest A leaves. There are two isoforms of BGLU in Arabidopsis. One of them, known as BG1 or BGLU18, is localized in the ER and undergoes dehydration-induced post-translational activation involving polymerization into high molecular weight complexes [26,64,65]. In contrast, the BG2 or BGLU33 isoform is localized in the vacuole, where under normal growth conditions it functions as an active, stable multimer whose level increases after dehydration [27]. Currently, there is no biochemical confirmation of which *BGLU* gene encodes the enzyme that can hydrolyze ABA-GE in pea plants. Therefore, to accurately assess the changes in the deconjugation of ABA-GE, it is essential to study the activity of the enzyme rather than focusing on the expression of the genes encoding it. In the oldest leaves, the observed increase in PsBGLU activity in response to Cd was the lowest compared to the other leaf developmental stages. Moreover, the expression of *PsABAUGT1* increased the most in the oldest leaves under Cd treatment, suggesting the formation of ABA-GE conjugates and consequently leading to a decrease in the concentration of free ABA in these leaves. In contrast, the expression of *PsABAUGT1* increased the least in the youngest leaves (Figure 3B), where the greatest increase in ABA-GE concentration was observed upon Cd treatment (Figure 1B). Therefore, based on the analysis of transcript levels, it is likely that ABA-GE is primarily synthesized in the oldest leaves in response to Cd stress and subsequently transported to the youngest leaves, where it undergoes hydrolysis to ABA.

### 3.3. The Expression of PYLs Increases Only in Young A and B1 Leaves under Cd Treatment

The phylogenetic relationship of the *PYLs* (Figure 4) revealed that the ABA receptors of Pisum sativum belong to the same subfamilies as those of *Arabidopsis thaliana*, *Citrus sinensis*, *Glycine max*, *Medicago truncatula*, *Oryza sativa*, *Triticum aestivum* or *Zea mays* [66]. *PsPYL8* and *PsPYL9* were assigned to subfamily I, *PsPYL4* to subfamily II and *PsPYL1* and *PsPYL2* to subfamily III (Figure 4). However, the expression of the *PYLs* of the individual subfamilies in response to stress factors is generally not related to phylogenetic relationships. The expression of each ABA receptor varies according to plant species, tissue type, type of stress, duration and intensity of stress, resulting in a different scenario under different stress conditions. For example, under salinity, the transcript level of *TaPYL1* is induced in roots while it decreases in shoots, while a decrease in the *TaPYL1* transcript level was observed after dehydration in both leaves and roots [67]. In roots of *Zea mays*, the expression of *ZmPYLs*, with the exception of *ZmPYL6*, was activated by osmotic stress induced by polyethylene glycol (PEG); however, the response was time-dependent [66]. Time course analyses showed that *ZmPYL8-9* are early response genes, and *ZmPYL1-7* and *ZmPYL10-11* are late response genes. In turn, in maize leaves *ZmPYL1-3*, *ZmPYL5-6* and *ZmPYL9-11* were down-regulated by dehydration, while *ZmPYL4* and *ZmPYL7-8* were significantly up-regulated. With the exception of *ZmPYL4*, which has been shown to belong to the group of late-responding genes together with *ZmPP2C* and *ZmSnRK2*, the other *ZmPYL* genes responded rapidly to water deprivation in maize leaves [68]. 

In pea plants, the expression of *PsPYLs* increases in response to cadmium in young A and B1 leaves and consistently decreases in mature B2 leaves and old C leaves, with the exception of *PsPYL1*, whose transcript level decreases in leaves at all developmental stages (Figure 5). The results indicate enhanced ABA perception in young leaves, where free ABA content was highest, suggesting a possible link between ABA content and perception mechanisms. In addition, the increased sensitivity to ABA could contribute to better protection of the youngest leaves. Similarly, in maize, sensitivity to herbivore-induced volatiles such as (Z)-3-hexenyl acetate varied with leaf age, resulting in high and persistent activation of jasmonate-mediated defenses exclusively in young leaves [69]. In older pea leaves, Cd-induced de novo ABA synthesis (Figure 2 and Figure 3) and ABA release from inactive conjugates are most pronounced, while the expression of *PsPYLs* genes is decreased (Figure 5). Therefore, the question arose whether a higher level of free ABA in older leaves would also increase the expression of *PsPYLs* as in A leaves. To clarify this uncertainty, plants were treated exogenously with 50 µM ABA for 24 h. It was found that the level of free ABA in the oldest C leaves increased approximately 7-fold after treatment, reaching a level almost equal to that of the youngest A leaves (Figure S3). Interestingly, the expression of all *PsPYLs* analyzed consistently decreased after ABA treatment at every developmental stage, including A leaves (Figure 6). The different expression profiles of *ZmPYL4* and *ZmPYL5* were also observed in maize roots and leaves, respectively, when the plants were exposed to ABA treatment and osmotic (PEG) or dehydration stress [68]. Interestingly, the transcript level of *OsPYL10* was down-regulated by abscisic acid in shoots and up-regulated in roots [70]. The observations made for *PsPYLs* may suggest that plants control the perception of ABA signaling to avoid undesirable effects, such as stunted growth, when ABA levels are too high. In this study, *PsPYL4* expression was found to be the most sensitive to changes in ABA concentration, while *PsPYL9* was the least sensitive. Studies in Arabidopsis and rice have shown that overexpression of *PYL9* genes promotes senescence of mature leaves in response to ABA treatment [37]. Therefore, it can be hypothesized that reduced *PsPYLs* transcription prevents the capture of de novo synthesized ABA before it is transported to the youngest leaves in the form of ABA-GE. Consequently, reducing the expression of *PYLs* may prevent the plant from triggering an exaggerated and prolonged stress response and premature senescence. However, further studies are needed to confirm this assumption.

### 3.4. Perspectives for the Development of ABA-Mediated Strategies to Increase Plant Resistance to Cadmium

The results of this study not only shed light on how leaves prioritize and control their responses to Cd stress at different developmental stages, but by identifying genes involved in the regulation of ABA content and ABA perception, they also open up possibilities for their application in the development of strategies to improve plant stress resistance. The possibility of using genes encoding enzymes involved in de novo ABA synthesis (*PsNCED2*, *PsAO3*) and release from conjugates with glucose (*PsBGLU*) to generate transgenic plants seems particularly promising. Although studies have already been conducted in which ABA metabolism has been modified to improve resistance of plants to Cd stress, there are still too few studies to fully understand the complex and interlinked molecular mechanisms behind this resistance. Transgenic lines of *Arabidopsis thaliana* obtained by ectopic expression of *Malus hupehensis NCED3* showed increased ABA accumulation, higher seed germination rate, better growth and lower water loss under Cd stress [71]. They also exhibited higher antioxidant enzyme activity, which was probably responsible for the reduction in oxidative damage and apoptosis compared to wild-type plants [71]. Moreover, ectopic expression of *MhNCED3* in transgenic *Arabidopsis thaliana* and apple calli showed reduced expression of genes related to Cd^2+^ uptake (*NRAMP*, natural resistance-associated macrophage protein, and *IRT*, iron-regulated transporter) as well as reduced Cd^2+^ influx and Cd content [13]. In turn, knocking down *BGLU10* or *BGLU18* reduced endogenous ABA levels, resulting in higher *NRT1.5* (nitrate transporter 1.5) levels and lower vacuolar H^+^-ATPase activity, which in turn led to higher Cd accumulation and sensitivity [72]. When generating transgenic plants with elevated ABA content, the possible undesirable physiological effects of these genetic modifications, such as growth inhibition, premature senescence or reduced photosynthetic efficiency due to prolonged stomatal closure, should also be considered. On the other hand, as highlighted in the discussion, the expression of genes encoding ABA receptors is regulated by the plant, presumably to avoid these undesirable physiological effects of long-term elevated ABA concentrations. In this way, the complex network that regulates the plant’s response to the current ABA concentration becomes as important as the ABA concentration itself and should also be taken into account when developing strategies to increase plant resistance to stress factors such as heavy metals.

## 4. Materials and Methods

### 4.1. Plant Material and Experimental Conditions

Seeds of *Pisum sativum* L. (cv. Iłówiecki) were germinated in moist vermiculite and 12-day-old seedlings were transferred to containers filled with aerated 1/2 Hoagland medium [73]. Nitrogen was added in the form of 4 mM NaNO_3_. After seven days, the medium was supplemented with CdCl_2_ (50 µM) or ABA (50 µM). Plants grown in a medium without CdCl_2_ and ABA served as a control. After 48 h of treatment, the plants (twelve plants per treatment) were harvested and separated into roots and leaves (leaflets plus stipule). The leaves were bulked according to their stage of development (Figure S1). The oldest true leaves were taken from the first and second node from the bottom and labeled as C leaves. Leaves from the third to fifth node were termed B2 leaves. The youngest fully expanded leaves were taken from the sixth node and labeled as B1 leaves, while the youngest unexpanded leaves were labeled as A leaves. Leaf samples were frozen in liquid N_2_ immediately after harvest and stored at −80 °C until use. 

The experiments were carried out in a growth chamber at a humidity of about 60% and light intensity of 250 µE m^−1^ s^−1^. The air temperature was 22 °C (16 h) during the day and 19 °C (8 h) at night. The experiment was repeated three times independently, resulting in three independent biological replicates.

### 4.2. Determination of ABA and Its Conjugates

Free and conjugated forms of ABA were extracted and determined as previously described by Sauter and Hartung [74], Dietz et al. [75] and Watanabe et al. [65] with some modifications. In brief, tissue pulverized in liquid nitrogen was extracted with 10 mM CaCl_2_ for 24 h in the dark at 4 °C. The ratio of tissue to buffer was 1:7 (*w*:*v*). The homogenate was centrifuged at 16,000× *g* for 30 min at 4 °C. The resulting supernatant was acidified with 0.1 M HCl to pH 3.0 and partitioned three times against equal volumes of ethyl acetate. The combined upper organic fractions containing free ABA were evaporated to dryness using a vacuum centrifuge and taken up in TBS buffer (Tris-buffered saline, 50 mM Tris-HCl, pH 7.8, 0.1 mM MgCl_2_ and 0.15 M NaCl). The lower aqueous fractions, containing ABA conjugates, were hydrolyzed with 1 M NaOH at 60 °C for 1 h in the dark. After alkaline hydrolysis, the samples were adjusted to pH 3.0 with 1 M HCl, partitioned three times against ethyl acetate, dried and taken up in TBS buffer. The levels of free ABA and ABA released from the conjugates were measured using the Phytodetek enzyme linked immunosorbent assay (ELISA) kit (Agdia, Inc., Elkhart, IN, USA) according to the protocol provided.

### 4.3. Determination of Aldehyde Oxidase Activity and Protein Content

Extraction of soluble plant proteins was performed in an ice-cold extraction medium containing 250 mM Tris–HCl, pH 8.5, 1 mM EDTA, 10 mM GSH and 2 mM DTT. A ratio of 1 g tissue to 3 mL buffer (1:3, *w*/*v*) was used. The homogenate was centrifuged at 16,000× *g* at 4 °C for 20 min. The resulting supernatant was subjected to native polyacrylamide gel electrophoresis (PAGE) on 7.5% separating gels in a Laemmli buffer system without SDS [76]. After electrophoresis, AO activity was stained as previously described [18] using indole-3-aldehyde as substrate. The relative intensity of the AO activity bands was quantified using NIH Image 1.62 software (Research Services Branch, National Institutes of Health). The total soluble protein content was determined using the Bradford method [77].

### 4.4. ABA-GE Hydrolyzing Activity Assay

The activity releasing free ABA from its glucose conjugates was determined as previously described [26,65] with slight modifications. In brief, 1 g of frozen tissue was homogenized with 5 mL of lysis buffer (25 mM HEPES, pH 7.0, 250 mM sucrose, 1 mM DTT, 10 mM MgCl_2_) and centrifuged at 10,000× *g* at 4 °C for 5 min to remove debris. Subsequently, the resulting supernatant was centrifuged at 100,000× *g* at 4 °C for 1 h. The microsomal fraction was then resuspended in 0.5 mL of lysis buffer containing 1% (*v*/*v*) Triton X-100 and incubated on ice for 30 min. Samples were then diluted 10-fold with lysis buffer, and 0.4 mL aliquots were then used to assay ABA-GE hydrolyzing activity in a 1 mL reaction mixture containing 100 pmol ABA-GE (OlChemim Ltd., Olomouc, Czech Republic) at 37 °C for 1 h. After incubation, the mixtures were diluted 20-fold in TBS buffer, and the amount of ABA released was determined by ELISA as described above. 

### 4.5. RNA Extraction and Gene Expression Analysis

Total RNA was isolated according to Chomczynski and Sacchi [78]. The isolated RNA was treated with DNase I and a total of 4 µg of RNA was reverse transcribed using the Transcriptor First Strand cDNA Synthesis Kit (Roche Diagnostics, Mannheim, Germany) according to the manufacturer’s protocol. Gene expression was analyzed by real-time PCR using a LightCycler^®^ 96 instrument (Roche Diagnostics, Mannheim, Germany). The reaction mixture contained 5 μL SYBR Green I Master Mix (Roche Diagnostics, Mannheim, Germany), 3 μL cDNA template (equivalent to 10 ng total RNA) and 0.5 μM of each primer in a total volume of 10 μL. The thermocycling conditions were as follows: 10 min at 95 °C, 40 cycles of 15 s at 95 °C, 15 s at 62 °C and 15 s at 72 °C. Melting curves (95 °C for 10 s, 65 °C for 1 min and 97 °C for 1 s) were generated for each reaction to ensure the specificity of amplification. The relative expression level of target genes was calculated using the 2^−ΔΔCt^ method [79]. The gene coding for actin was used as an internal control. All primers used for real-time PCR are listed in Table S1. All reactions were performed in at least triplicate for each of the three biological replicates.

### 4.6. Bioinformatics Analysis of PYL Sequences

A database search of the National Center for Biotechnology Information (http://www.ncbi.nlm.nih.gov; accessed on 3 February 2023) for *Pisum sativum* PYL-like sequences was performed using the BLAST algorithm. The maximum likelihood tree was constructed using the program PhyML 3.0 and the Shimodaira-Hasegawa-like (SH-like) approximate likelihood ratio test [80].

### 4.7. Statistical Analyses

Statistical analyses were based on three independent experiments (biological replicates) with three technical replicates for each experiment and are presented as mean  ±  SD. Data were analyzed by a two-way analysis of variance (ANOVA) using leaf development stage and treatment as two parameters. The conformity of the data to a normal distribution was checked using the Shapiro–Wilk test, and the homogeneity of variances in the data sets was confirmed using the Levene test. The significance of the differences between the group means was assessed using the parametric Tukey test. All statistical analyses were performed using Statistica ver. 9.1 software.

## Data Availability

Data are contained within the article and Supplementary Materials.

## Supplementary Materials

The following supporting information can be downloaded at: https://www.mdpi.com/article/10.3390/ijms25126582/s1.
