# Supplementary material for: Cadmium Alters the Metabolism and Perception of Abscisic Acid in Pisum sativum Leaves in a Developmentally Specific Manner
Source: Int J Mol Sci. 2024 Jun 14;25(12):6582. doi: 10.3390/ijms25126582 (PMC11203977; doi:10.3390/ijms25126582)
Supplement: Supplementary file 1 [file ijms-25-06582-s001.zip › ijms-3033414-supplementary.pdf]

Cadmium alters the metabolism and perception of abscisic acid in *Pisum sativum* leaves in a developmentally specific manner

Edyta Zdunek-Zastocka*, Beata Michniewska, Angelika Pawlicka, Agnieszka Grabowska

Department of Biochemistry and Microbiology, Warsaw University of Life Sciences – SGGW, Nowoursynowska 159, 02-776 Warsaw, Poland;
* Correspondence: edyta_zdunek_zastocka@sggw.edu.pl Tel.: +48 22 593 2577;

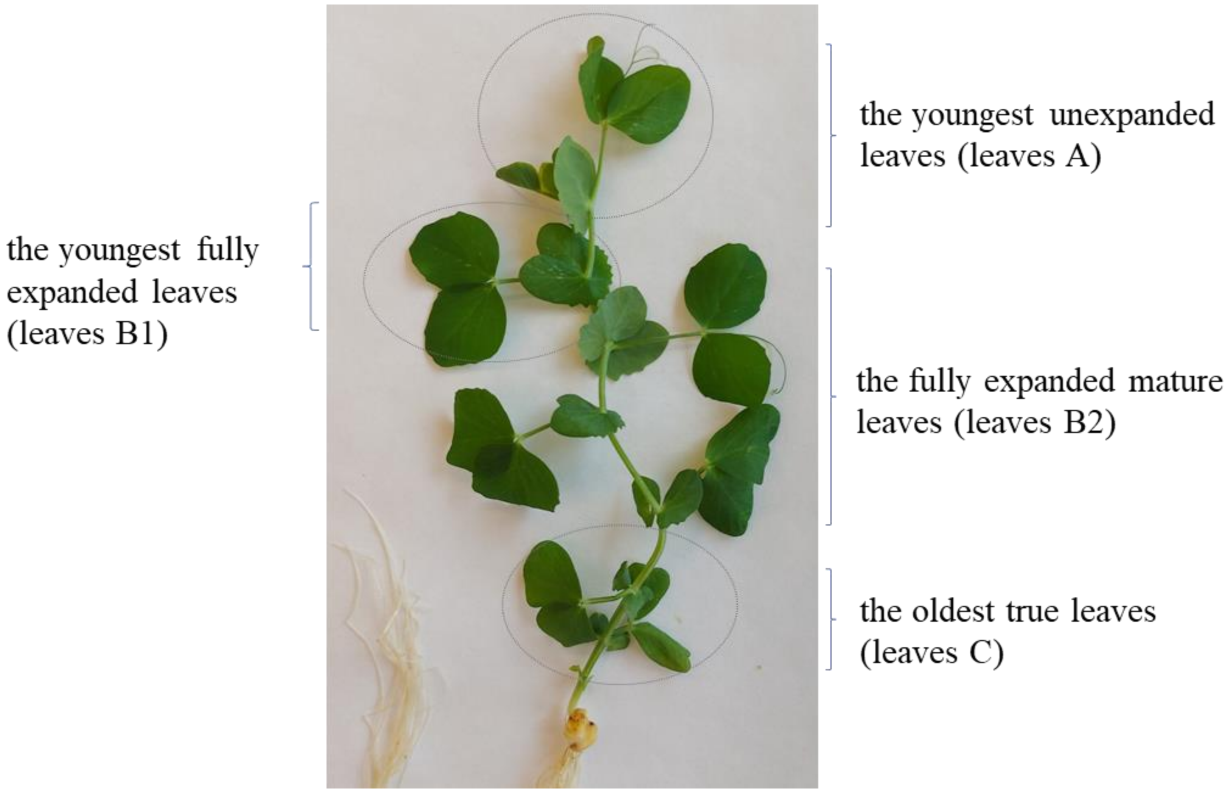

Figure S1. Morphology of 21-days old *P. sativum* plants grown on 1/2 Hoagland medium without $CdCl_2$ and ABA.

Table S1. Primer sequences used for real-time PCR analysis of the expression of *Pisum sativum* genes. F, forward oligonucleotide; R, reverse oligonucleotide.

**Gene**	**Accesion number of the sequence in GenBank database**	**Oligonucleotide sequence (5'->3')**
PsNCED2	AB080192	F: TGACATCCCATATCACGTTCGTG<br>R: CCATGGCTCAGCAAGAGCGAG
PsNCED3	AB080193	F: CATGGTCACTCGGGTATCGCAAG<br>R: AACCTTCCATCACCGTAGCTATG
PsAO3	EF491600	F: CAGCTGGATGGAGTAAGAGATG<br>R: GTGCTGTAACTATGTTATGTGG
PsABAUGT1	MF034884	F: GTAGCTTGCTTACCGATGAAACAGCTC<br>R: CCAATTTTCACTGCAAGTTTGATCATCAC
PsPYL1	XM_051050039	F: AACCGCAAATCTACAAACACTTCATC<br>R: ACATTGACGTCTCTGGTGCAAC
PsPYL2	XM_051029745	F: GGAACACTGAAGAAGATACCAAGATG<br>R: AGCAACAACTCCAAGTTTCTGAAG
PsPYL4	XM_051056584	F: CTAGCTAACTACCGTTCTGTCACC<br>R: CAACCACGTAAGACTCAACCAAC
PsPYL8	XM_051037273	F: GTTGGAGCTTCTTGATGACAATGAG<br>R: GAGTAGTTCGTAAGCCTATGATCAC
PsPYL9	XM_051017615	F: AGGTGATCTTGGCATTGGAAGTG<br>R: CTCAGTACTAGTTGTAGCTGGAAGACCAG
Actin	X90378	F: ATCATGGAGCCTGAGAGTTG<br>R: CACATACCACTAGGCCAATC

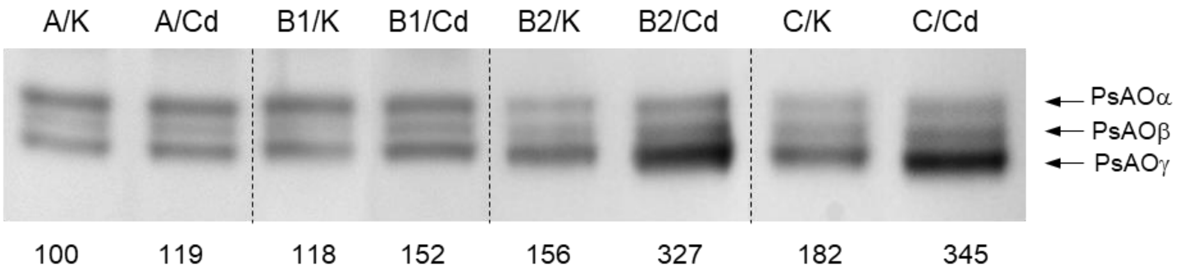

Figure S2. Zymograms of AO in the leaves of the pea plants after 48 hours of Cd treatment. Cadmium was applied as 50 µM $CdCl_2$. AO activity was assayed after native PAGE with indole-3-aldehyde as a substrate. Each lane of the gel was loaded with 100 µg of proteins. The zymograms are representative of similar results obtained in three independent biological experiments. A, the youngest unexpanded leaves; B1, the youngest fully expanded leaves; B2, fully expanded mature leaves; C, the oldest leaves; K – control conditions, Cd – cadmium applied as 50 µM $CdCl_2$.

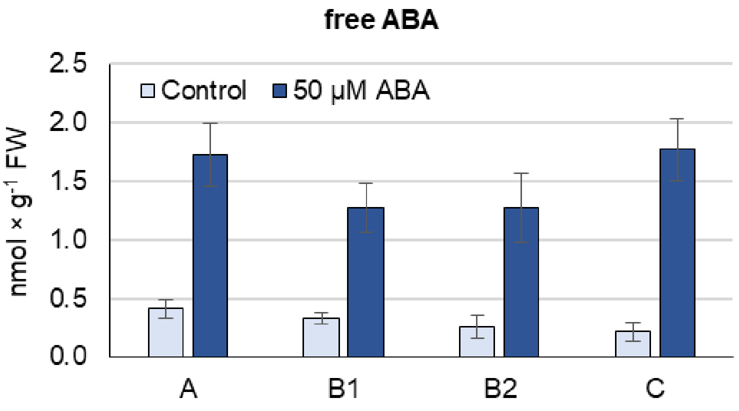

Figure S3. Changes in the abscisic acid content after 24 hours of treatment with abscisic acid. ABA was applied at concentrations of 50 µM. ABA was extracted and determined as described by Zdunek-Zastocka et al. (2013). A, the youngest unexpanded leaves; B1, the youngest fully expanded leaves; B2, fully expanded mature leaves; C, the oldest true leaves. The results are the means (±SD) of three biological replicates. Asterisks indicate statistically significant differences between control and ABA-treatment ($P < 0.05$).

References

1. E. Zdunek-Zastocka, M. Sobczak, Expression of *Pisum sativum PsAO3* gene, which encodes an aldehyde oxidase utilizing abscisic aldehyde, is induced under progressively but not rapidly imposed drought stress. Plant Physiol Biochem. 71 (2013) 57–66.
